# Resolvin D1/N-formyl peptide receptor 2 ameliorates paclitaxel-induced neuropathic pain through the activation of IL-10/Nrf2/HO-1 pathway in mice

**DOI:** 10.3389/fimmu.2023.1091753

**Published:** 2023-03-13

**Authors:** Cun-Jin Su, Jiang-Tao Zhang, Feng-Lun Zhao, De-Lai Xu, Jie Pan, Tong Liu

**Affiliations:** ^1^ Institute of Pain Medicine and Special Environmental Medicine, Nantong University, Nantong, China; ^2^ Department of Pharmacy, The Second Affiliated Hospital of Soochow University, Suzhou, China; ^3^ College of Life Sciences, Yanan University, Yanan, China; ^4^ Suzhou Key Laboratory of Intelligent Medicine and Equipment, Suzhou Medical College of Soochow University, Suzhou, China

**Keywords:** RvD1, paclitaxel, FPR2, Il-10, macrophage

## Abstract

**Introduction:**

Paclitaxel is a chemotherapy drug that is commonly used to treat cancer, but it can cause paclitaxel-induced neuropathic pain (PINP) as a side effect. Resolvin D1 (RvD1) has been shown to be effective in promoting the resolution of inflammation and chronic pain. In this study, we evaluated the effects of RvD1 on PINP and its underlying mechanisms in mice.

**Methods:**

Behavioral analysis was used to assess the establishment of the PINP mouse model and to test the effects of RvD1 or other formulations on mouse pain behavior. Quantitative real-time polymerase chain reaction analysis was employed to detect the impact of RvD1 on 12/15 Lox, FPR2, and neuroinflammation in PTX-induced DRG neurons. Western blot analysis was used to examine the effects of RvD1 on FPR2, Nrf2, and HO-1 expression in DRG induced by PTX. TUNEL staining was used to detect the apoptosis of DRG neurons induced by BMDM conditioned medium. H2DCF-DA staining was used to detect the reactive oxygen species level of DRG neurons in the presence of PTX or RvD1+PTX treated BMDMs CM.

**Results:**

Expression of 12/15-Lox was decreased in the sciatic nerve and DRG of mice with PINP, suggesting a potential involvement of RvD1 in the resolution of PINP. Intraperitoneal injection of RvD1 promoted pain resolution of PINP in mice. Intrathecal injection of PTX-treated BMDMs induced mechanical pain hypersensitivity in naïve mice, while pretreatment of RvD1 in BMDMs prevented it. Macrophage infiltration increased in the DRGs of PINP mice, but it was not affected by RvD1 treatment. RvD1 increased IL-10 expression in the DRGs and macrophages, while IL-10 neutralizing antibody abolished the analgesic effect of RvD1 on PINP. The effects of RvD1 in promoting IL-10 production were also inhibited by N-formyl peptide receptor 2 (FPR2) antagonist. The primary cultured DRG neurons apoptosis increased after stimulation with condition medium of PTX-treated BMDMs, but decreased after pretreatment with RvD1 in BMDMs. Finally, Nrf2-HO1 signaling was additionally activated in DRG neurons after stimulation with condition medium of RvD1+PTX-treated BMDMs, but these effects were abolished by FPR2 blocker or IL-10 neutralizing antibody.

**Discussion:**

In conclusion, this study provides evidence that RvD1 may be a potential therapeutic strategy for the clinical treatment of PINP. RvD1/FPR2 upregulates IL-10 in macrophages under PINP condition, and then IL-10 activates the Nrf2- HO1 pathway in DRG neurons, relieve neuronal damage and PINP.

## 1 Introduction

Paclitaxel (PTX) is widely used chemotherapy for the treatment of a range of malignancies, including breast, ovarian and lung cancer (1). However, PTX often produces peripheral neuropathy and neuropathic pain in the distal extremities, persisting for months or years (2). PTX-induced peripheral neuropathic pain (PINP) is a serious dose-limiting adverse effect during PTX treatment in cancer patients (3). PINP greatly decreased the life quality of patients during and after chemotherapy. Because of PINP, life-saving cancer treatment usually has to be discontinued. To date, there are no Food and Drug Administration-approved drugs to prevent or treat PINP. Accordingly, there is an urgent need to develop novel therapies for preventing or treating PINP, improving both cancer treatment and life quality of afflicted patients.

Mounting evidences indicate that macrophages contribute to initiation, maintenance, and resolution of chronic pain through neuro-immune interactions (4). Under neuronal injury conditions, the injury neurons and resident macrophages are able to produce pro-inflammatory factors, chemokines and damage-associated molecular patterns (DAMPs), form a local inflammatory microenvironment, and further recruit circulating macrophages to infiltrate peripheral nerve tissue (5, 6). The dorsal root ganglion (DRG), a structure that transmits pain signals from the periphery to the central nervous system, plays an important role in the development and maintenance of chronic pain (7). A marked infiltration of macrophages was demonstrated in the DRGs of peripheral neuropathic pain model animals induced by multiple chemotherapeutic agents, including paclitaxel, platinum, and vincristine (8). Classically, macrophages are subdivided into two major phenotypes, M1-like and M2-like. M1-like macrophages are pro-inflammatory, and M2-like macrophages are anti-inflammatory. Long-term activation of M1-like macrophages is considered to be important mechanisms for the chronic neuropathic pain, possible by activating the TRP channels and mitogen-activated protein kinase (MAPK) signaling (9). M2-like macrophages can secrete a variety of anti-inflammatory factors, including IL-4, IL-10, TGF-β, inhibit local inflammatory response, and alleviate neuropathic pain (10). It is worth noting that inhibition of the inflammatory response of macrophages or deleting macrophages with drugs can relieve pain in animal models of peripheral neuropathic pain (11, 12). Collectively, these studies suggest that targeting macrophages may be an important strategy for alleviating neuropathic pain (13).

Resolvins are endogenous mediators that promote the inflammation resolution and are able to return the inflamed tissues to homeostasis (14). Resolvin D1 (RvD1), a member of resolvins family, is mainly detected in macrophages and neutrophils (15). RvD1 can enhance the phagocytosis of macrophages, enhance the clearance of aging cells, and promote macrophages to produce IL-10 (16, 17). In our previous study, we demonstrated that RvD1 or RvE1 attenuated formalin-induced inflammatory pain in mice (18). Although RvD1 is well-known to regulate macrophage function, it is unclear whether it can alleviate PINP through acting on macrophages. In the present study, we tested the hypothesis that RvD1 attenuates PINP possible through the regulation of macrophages in the DRGs.

## 2 Materials and methods

### 2.1 Animals

Male ICR mice (8-10 weeks of age) from SLAC Company (Shanghai, China) were used in this study. Mice were housed four per cage with free access to food and water. All mice were kept in controlled room temperature (22 ± 2°C) and humidity (60-80%). The illumination maintained on a 12h/12h light/dark cycle (lights on from 6:00 AM to 6:00 PM). Mice were numbered according to body weight, and then grouped by looking up the random number table. The number of mice in each group was shown in the corresponding figure legends. All animal experiments were blind to the operators during the allocation, the conduct of the experiment, the outcome assessment. All animal experiments and procedures were performed in accordance with the guidelines recommended by the International Association for the Study of Pain, and were approved by the University Committee on Animal Care of Soochow University.

### 2.2 Paclitaxel-induced neuropathic pain model

Paclitaxel (PTX) solution (Yangtze River Pharmaceutical Group, China) was diluted with saline before treatment. PTX was injected intraperiotoneally (i.p) in mice at a dose of 2 mg/kg on days 0, 2, 4 and 6 to generate PTX-induced neuropathic pain. The final PTX cumulative dose was 8 mg/kg per mouse. Mice in the control group received an intraperitoneal injection of saline as vehicle.

### 2.3 Drug administration

Mice received intraperitoneal　(i.p.)　injection of RvD1 (Cayman Chmeical, USA) at a dose of 5 μg/kg for 14 days or for a single injection.

### 2.4 Intrathecal injection

Mice were under a brief anesthesia with isoflurane, then we delivered drugs into cerebral spinal fluid (CSF) space around lumbosacral spinal cord through intrathecal (i.t.) injection. Spinal cord puncture was made with a 30G needle between the lumbar L5 and L6 to inject the IL-10 neutralizing antibody (Sigma, I5145, 10ug/10ul) to the CSF. A brisk tail-flick after the needle entry into subarachnoid space signed a successful spinal puncture.

### 2.5 Behavioral tests

Behavioral tests were performed in a quiet and temperature-controlled room between 9:00 AM and 5:00 PM, and were carried out by an operator blinded to drug treatments.

Mechanical allodynia: Mechanical allodynia was assessed using the “up-and-down” methods as previously described (19). Mice were placed beneath perspex boxes (10×10×7cm) set upon elevated wire mesh stands and acclimated for 30 min. Von Frey filament (0.008-1.4g) was applied to the mid-plantar area (avoiding the base of tori) with enough pressure to bend the hair. The filament was held for 5s. If the paw did not lift after 5s, an increased weight filament would be used next. Whereas, a subsequently weaker filament would be used if the paw lifted after filament stimuli. The 50% mechanical paw withdrawal threshold was calculated as described previously (19). The paw mechanical withdraw thresholds were recorded in grams (g).

Thermal hyperalgesia: The thermal hyperalgesia was measured using tail flick test. Tail flick test was carried out by immersing the mouse tail in water (5 cm from the tip) maintained at 48°C.Tail flick latency time was measured as the time from the heat exposure to the withdraw of the tail. The cut off time for tail flick test was 15s. This test was carried out 3 times per mouse, and the average value was taken as latency time.

Cold hyperalgesia test: Cold hyperalgesia was analyzed by the acetone stimulation test. Mice were placed into perspex boxes (10×10×7cm) with a wire mesh floor. Mice were allowed to habituate for 30min prior to the test. A drop (50 μl) of acetone was placed onto the center of planta skin. The responses to acetone were recorded in the following 30s after acetone application. Responses to acetone are divided into 4 grades: 0, no response; 1, quick withdraw, flick or stamp of the paw; 2, prolonged withdraw or repeat flicking of the paw; 3, repeated flicking of the paw with licking directed at the ventral side of the paw. Acetone was applied to each paw 3 times at a 10-15 min intervals, and the average score was calculated.

### 2.6 Cell culture

Bone marrow-derived macrophages (BMDMs) were isolated from the tibia and femoral bone marrow of 8-week-old male mice. BMDMs were cultured in Dulbecco’s modified Eagle’s medium (DMEM) containing 10% fetal bovine serum, 20 ng/ml recombinant murine GM-CSF (novoprotein) and 1% penicillin/streptomycin. The DRG tissue of 2-3 week old mice was isolated, digested with 0.15% collagenase and 0.25% trypsin until there was no visible tissue mass. After being filtered by 70 μm filter membrane, centrifuged and cultured in the neurobasal medium containing 10% serum, 2% B27 and 1% penicillin/streptomycin. Cells were cultured as a monolayer under 5% CO2 in a humidified incubator at 37°C.

### 2.7 Quantitative real-time polymerase chain reaction

Total RNA was extracted using Trizol Reagent (Invitrogen, Carlsbad, CA) according to the protocol supplied by the manufacturer. The RNA amount and quality were assessed by Microplate Reader (Thermo). RNA (500 ng) was converted to cDNA using RevertAid First Strand cDNA Synthesis Kit (Thermo Fisher Scientific, Waltham, USA). Real-time PCR was conducted using SYBR Green PCR Master Mix (Selleck, China) on Opticon real-time PCR Detection System (Applied Biosystems 7500, Grand Island, NY). Relative fold of differences in expression were calculated using the 2(-Delta Delta C(t)) method after normalization to GAPDH expression. The following primers for mouse were synthesized by Genewiz: GAPDH (forward: 5’-GAAGGTCGGTGTGAACGGAT-3’; reverse: 5’-AATCTCCACTTTGCCACTGC-3’), FPR2 (forward: 5’-GTCAAGATCAACAGAAGAAACC-3’; reverse: 5’-GGGCTCTCTCAAGACTATAAGG-3’), 12/15-Lox (forward: 5’-GCGACGCTGC CCAATCCTAATC-3’; reverse: 5’-CATATGGCCACGCTGTTTTCTACC-3’), IL-4 (forward: 5’-ATGGATGTGCCAAACGTCCT-3’; reverse: 5’-CATATGGCCACGCT GTTTTCTACC-3’), IL-10 (forward: 5’-GGACTTTAAGGGTTACTTGGGTTGCC-3’; reverse: 5’-CATTTTGATCATCATGTATGCTTCT-3’), IL-1β (forward: 5’-TGTAA TGAAAGACGGCACACC-3’; reverse: 5’-TCTTCTTTGGGTATTGCTTGG-3’), TNF-α (forward: 5’-AGCCGATGGGTTGTACCTTG-3’; reverse: 5’-TTGGGCAGAT TGACCTCAGC-3’), TGF-β (forward: 5’-TGAACCAAGGAGACGGAATACAGG-3’; reverse: 5’-TACTGTGTGTCCAGGCTCCAAATG-3’).

### 2.8 Western blotting

Cells were washed 3 times with ice-cold PBS and lysed in RIPA supplemented with phenylmethylsulfonyl fluoride on ice for 30min. The samples were centrifuged at 12000rpm for 25min at 4°C, then the supernatant was collected. The protein concentration was assessed using a BCA protein assay (Beyotime, China). The protein samples were separated with 10% resolving gel and electroblotted onto a polyvinylidene fluoride (PVDF) membrane. The membranes were blocked with 5% non-fat milk for 1 h at room temperature, then incubated overnight at 4°C with primary antibodies (FPR2, Thermo Fisher, 720293; Nrf2, Proteintech, 16396-1-AP; HO-1, Proteintech, 10701-1-AP; Tubulin, Proteintech, 11224-1-AP; H3, Beyotime, AF7014), followed by HRP-conjugated secondary antibodies for 1 h at room temperature. Immunoreactive bands were detected by enhanced chemiluminescence (ECL) reagent using a chemiluminescence instrument. Image J was used to measure the grey intensity of the specific bands.

### 2.9 Immunohistochemistry

Mice were anesthetized using pentobarbital, and perfused through the ascending aorta with 4% paraformaldehyde. L4 and L5 DRGs were removed and postfixed in 4% paraformaldehyde overnight followed by dehydrating with 30% sucrose solution in PBS for 3 days. Cryostat sections (15 μm) were cut and stained for IHC with primary antibody (CD68, Abcam) overnight at 4°C. Then the sections were incubated with goat anti-Rat IgG - H&L (Alexa Fluor 488) for 1 h at RT.

### 2.10 TUNEL staining

Neuronal apoptosis was detected by the TdT (terminal deoxyribonucleotidyl transferase)-mediated dUTP nick-end labeling (TUNEL) assay (Beyotime, China). Briefly, primary DRG neurons were washed twice with PBS, fixed with 4% paraformaldehyde for 15 min, and then washed twice with PBS. TUNEL staining solution was prepared according to the instruction, dropped on the cells, reacted at 37°C for 1 hour, washed with PBS and then treated with DAPI staining solution for 3 minutes, washed with PBS for 3 times, and photographed with a microscope.

### 2.11 Detection of intracellular reactive oxygen species

The intracellular ROS of primary DRG neurons was detected by H2DCF-DA following the reference. After treatment, the DRG neurons were incubated with 25 mM H2DCF-DA for 30 min. After washing thrice with cold PBS, the cell images were acquired immediately *via* fluorescence microscopy.

### 2.12 Statistical analysis

The data were presented as the mean ± standard error of the mean (SEM). Student’s *t*-test was used to compare two groups, and one-way analysis of variance (ANOVA) was used to compare multiple samples. All statistical analyses were performed using the SPSS 13.0 software. P<0.05 is considered as statistically significant.

## 3 Results

### 3.1 PTX treatment induces PINP and impairs RvD1 synthesis in mice

**Figure 1:**
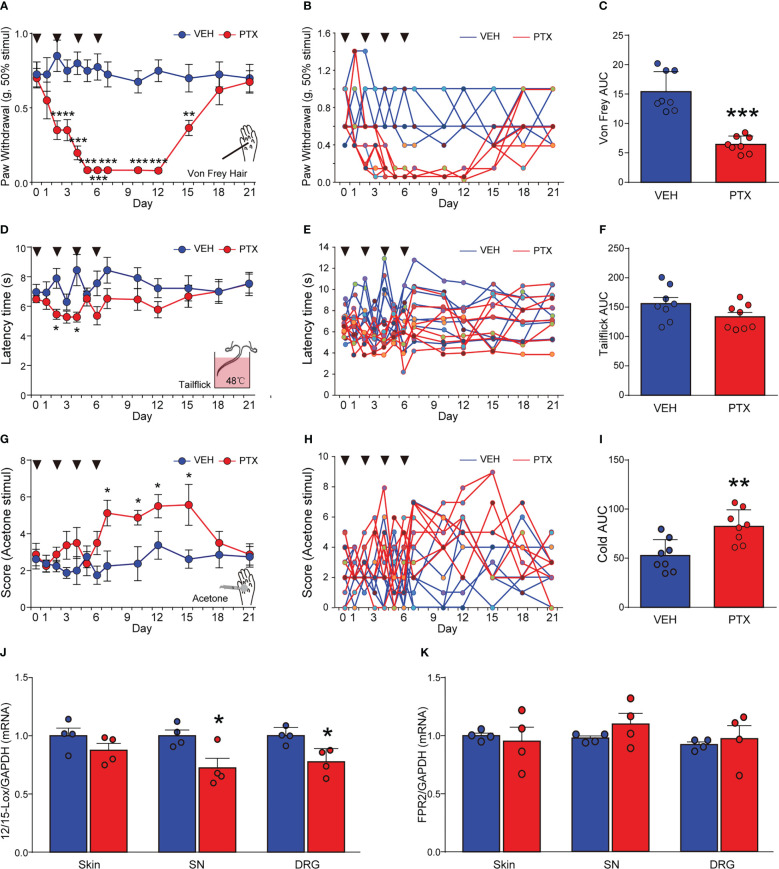
Paclitaxel induced peripheral neuropathic pain in mice, and decreased 12/15-Lox expression. PTX was injected intraperiotoneally (i.p) in mice at a dose of 2 mg/kg on days 0, 2, 4 and 6 to generate PTX-induced neuropathic pain. Nociceptive thresholds to mechanical **(A)**, heat **(D)** and cold **(G)** stimulation of the hindpaws of mice (**** means ** Day 2 and Day 3 vs VEH, ****** means *** Day 5, 7, 10, 12 vs VEH). Individual mouse time course plots showing changes in mechanical **(B)**, heat **(E)**, and cold **(H)**. Area under the time course cures (AUC) of mechanical **(C)**, heat **(F)**, and cold **(I)** thresholds. 12/15-Lox **(J)** and FPR2 **(K)** mRNAs in DRG, SN, skin tissues were determined by qPCR. *p < 0.05, **p < 0.01, ***p < 0.001 vs VEH group, n=8 **(A–I)**, n=4 **(J, K)**. PTX, paclitaxel; VEH, vehicle; AUC, area under curve.

PTX is a commonly used chemotherapy drug, but PINP is a common dose-limiting adverse drug reaction. PINP commonly exhibits an increased sensitivity to mechanical, heat and cold stimulation. Altered nociceptive thresholds to mechanical, heat and cold stimulation are hallmarks of the development of PINP. Adult male ICR mice received 4 injections of PTX (2 mg/kg, every other day, i.p.) for a total cumulative dose of 8 mg/kg. We assessed mechanical sensitivity in PTX-treated mice using von Frey filaments. PTX significantly reduced mechanical threshold to elicit a paw withdraw response. Increased mechanical sensitivity developed within 2 days post-initiation of PTX administration and was sustained for about 2 weeks (**Figures 1A–C**). The peak effects of mechanical hypersensitivity occurred between days 5 and 12 (**Figure 1A**). Thermal pain was detected by tail flick test at 48°C hot water. PTX-treated mice developed a slight and transient hypoalgesia to heat stimuli, which was observed on day 2 and 5 (**Figures 1D–F**). In contrast, PTX-treated mice were sensitive to cold stimuli. The acetone cold pain induced by PTX started on the 7^th^ day and continued until the 15^th^ day (**Figures 1G–I**). Together, these data indicated that mechanical and cold allodynia were successfully induced in PTX-treated mice (but not thermal hyperalgesia), and this phenotype was consistent with other reports (20).

Several studies reported that RvD1 could attenuate diverse inflammatory diseases (14), but the impact of RvD1 on PINP is still unclear. Herein, we detected 12/15-Lox (a synthase of RvD1) and FPR2 (a receptor for RvD1) mRNA levels in sciatic nerve (SN) and dorsal root ganglia (DRG) tissues using qPCR analysis at day 14 after PTX administration. It was found that PTX treatment significantly reduced 12/15-Lox mRNA expression by ~20% in SN and DRG tissues, but not in the skin from mice hindpaws (**Figure 1J**, p < 0.05). In contrast, PTX had no impact on FPR2 mRNA expression in SN, DRG and skin tissues (**Figure 1K**). The data indicated that RvD1 synthesis was impaired in the peripheral nervous system in PINP mice.

### 3.2 Systemic administration of RvD1 reduces PTX-induced mechanical and cold hyperalgesia

**Figure 2:**
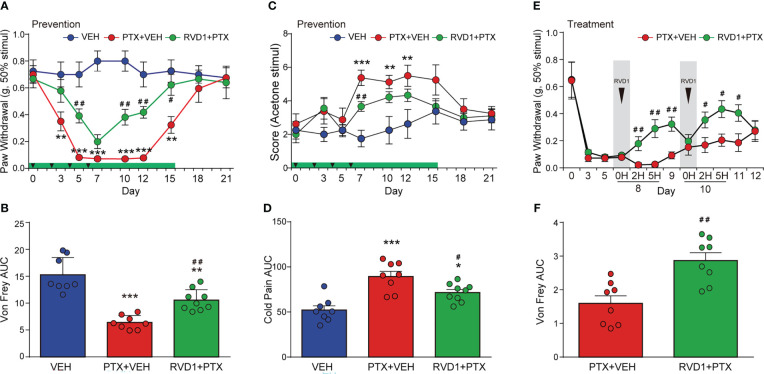
Systemic administration of RvD1 reduces PTX-induced mechanical and cold hyperalgesia. Mice were injected with RvD1 (5 μg/kg, ip) 1 day before PTX injection, and injected continuously for 15 days. mechanical pain threshold tested using Von Frey and AUC **(A, B)**, cold pain score tested using acetone stimulation and AUC **(C, D)**; single injection of RvD1 (5 μg/kg, ip) on the 8th and 10th days after the first PTX injection, mechanical pain threshold and AUC of the hind paw of mice **(E, F)**. *P < 0.05, **P < 0.01, ***P < 0.001 vs VEH group; #P < 0.05, ##P < 0.01 vs PTX group. n=8 in VEH and PTX groups, n=9 in RVD1+PTX group **(A–D)**; n=8 in each group **(E, F)**. RvD1, resolvin D1; PTX, paclitaxel; VEH, vehicle.

Next, we evaluated whether RvD1 could reduce mechanical and cold hyperalgesia induced by PTX in mice. First, we investigated the preventive efficacy of RvD1 on the development of PTX-induced pain hypersensitivity in mice. Mice received intraperitoneal (i.p.) injection of RvD1 (5 μg/kg/day) 24 h prior to PTX administration for a total of 15 days. RvD1 significantly reduced mechanical allodynia on day 5 after the first PTX injection (**Figure 2A**, p < 0.01). In RvD1 prevention group, the threshold of mechanical stimuli returned to baseline 15 days post-initiation of PTX administration, but it taken 18 days to recover without RvD1 prevention treatment (**Figure 2A**). Meanwhile, the area under curve (AUC) of Von Frey analysis also showed that RvD1 significantly reliefed the mechanical allodynia induced by RTX in mice (**Figure 2B**, p<0.01). RvD1 significantly attenuated cold allodynia only on day 7 compared with PTX-treated mice (**Figure 2C**, p < 0.01). Although this effect was less pronounced than that of mechanical allodynia, the area under curve (AUC) of cold pain analysis showed RvD1 significantly decreased PTX-induced cold pain in mice (**Figure 2D**, p < 0.05). These data indicated that preventive application of RvD1 not only attenuated mechanical and cold hyperalgesia induced by PTX in mice.

In addition, to investigate whether RvD1 could relieve pain when PINP was already established, we examined the potential therapeutic effect of RvD1 on PINP. An i.p. injection of RvD1 was given on day 8 following the first PTX injection. The mechanical allodynia was significantly inhibited at 2 h after RvD1 injection (**Figure 2E**, p < 0.01). This analgesic effect of RvD1 lasted for at least 2 days following RvD1 administration (**Figure 2E**). In addition, RvD1 still exhibited relief of mechanical pain with a single injection on day 10 (**Figure 2E**). Moreover, the area under curve of the Von Frey analysis also showed that the overall treatment with RvD1 significantly reliefed PINP (**Figure 2F**, P<0.01). These data indicated that RvD1 may be useful not only for prevention of PINP, but also for treatment of PINP.

### 3.3 RvD1 attenuates mechanical allodynia induced by PTX *via* regulating macrophages

**Figure 3:**
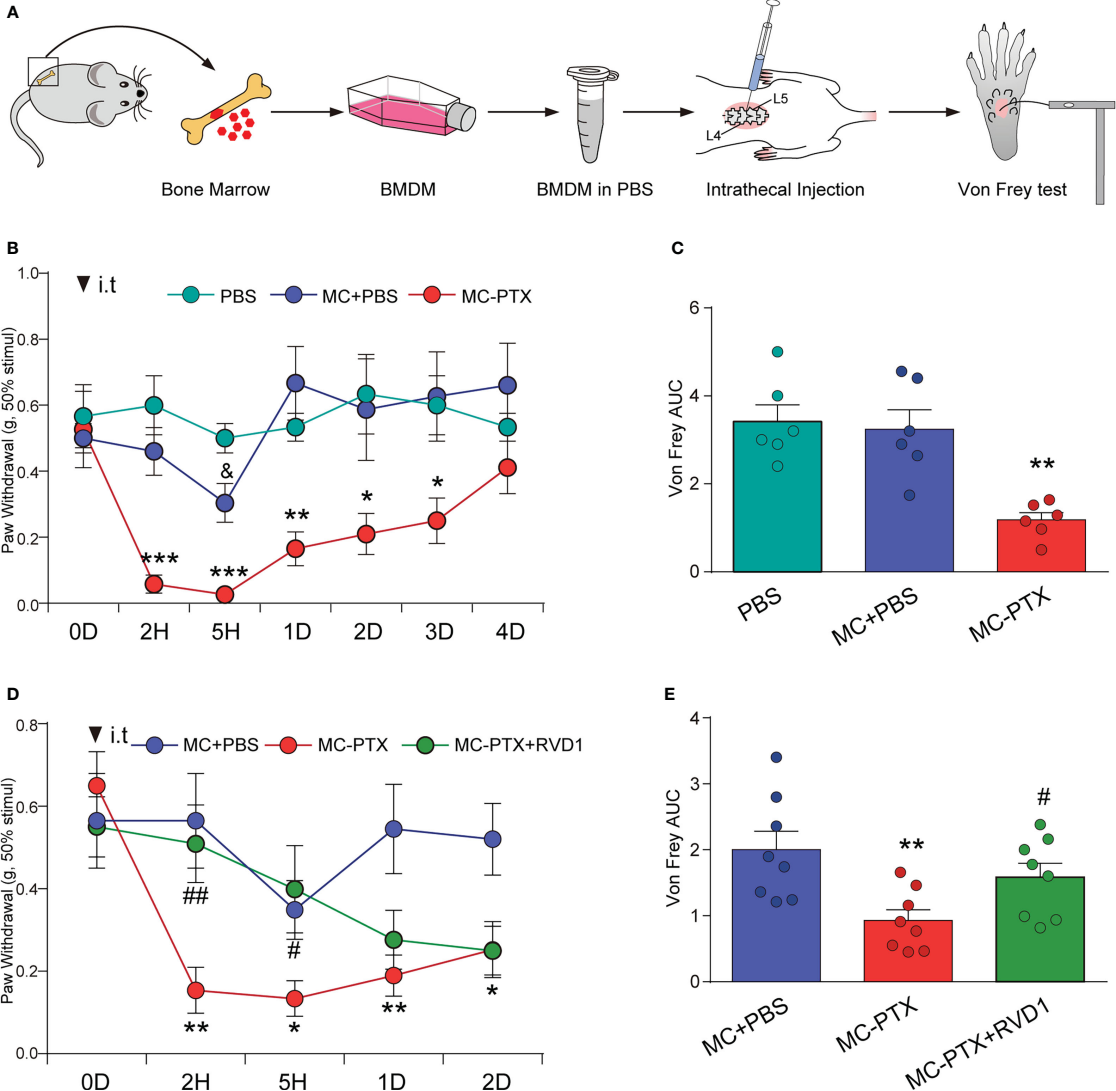
RvD1 attenuates mechanical allodynia induced by PTX *via* regulating macrophages. Mouse bone marrow-derived macrophages (BMDMs) were pretreated with 250 nm RVD1 for 3h, and then treated with 1 μM PTX for 24h. BMDMs of different treatment groups (3×10^3^) were intrathecally injected into mice intervertebral foramen. The process is shown schematically in **(A)**. Mechanical pain threshold was determined using Von Frey test **(B, D)**, AUC analysis was shown in **(C, E)**. *p < 0.05, **p < 0.01, ***p < 0.001 vs normal MC group; #p < 0.05, ##p < 0.01 vs MC-PTX group. n=6 in each group **(B, C)**; n=8 in each group **(D, E)**. MC, macrophage; RvD1, resolvin D1; PTX, paclitaxel.

Recent findings indicate that the immune system, especially macrophages, plays a critical role in the development and maintenance of chemotherapy-induced peripheral neuropathy (CIPN). To test the hypothesis that macrophages contribute to the behavior signs of PINP, we intrathecally (i.t.) administrated PTX-treated BMDMs into spinal cerebrospinal fluid *via* lumbar puncture, and tested the mechanical pain threshold using Von Frey hairs. The experimental procedure was shown in **Figure 3A**. BMDMs (3.0×10^3^ cells) were prepared in 10 μl PBS and collected for i.t. injection. As shown in **Figures 3B, C**, a single i.t. injection of normal BMDMs developed a rapid and transient (within 5 h) mechanical allodynia compared with PBS i.t. injection mice (p < 0.05). However, a single i.t. injection of PTX-treated BMDMs produced a remarkable decreased threshold of mechanical stimuli within 2 h (**Figure 3B**, p < 0.001). Notably, this nociceptive effect induced by PTX-treated BMDMs after i.t. injection lasted for 3 days (**Figure 3B**).

We next examined the effect of BMDMs pretreated with RvD1 followed by PTX treatment on mechanical allodynia. BMDMs were pretreated with RvD1 (250 nM) for 24 h and then stimulated with PTX (1 μM) for 24 h. As shown in **Figures 3D, E**, the threshold for mechanical stimuli in RvD1+PTX-treated BMDMs group maintained at the same level compared with normal BMDMs group within 5 h after i.t. injection, and was significantly higher than PTX-treated BMDMs group (p < 0.05). These data indicated RvD1 alleviated PINP by acting on macrophages.

**Figure 4:**
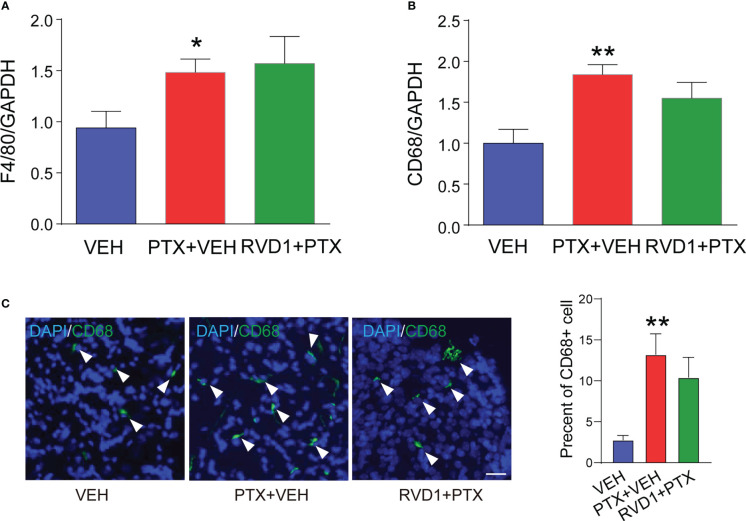
RvD1 did not affect the infiltration of macrophages in DRG under PINP condition. The mRNA of mouse DRG tissue was extracted 14 days after PTX injection. The expression of macrophage markers F4/80 **(A)** and CD68 **(B)** mRNA was detected by qPCR, and the CD68 positive cells **(C)** in DRG were detected by immunofluorescence technique. *p < 0.05, **p < 0.01 vs CON group. n=4. Scale bar: 50 μm. RvD1, resolvin D1; PTX, paclitaxel.

We further detected the expressions of macrophage markers in DRG and evaluated the effect of RvD1 on macrophage infiltration. First, we detected F4/80 and CD68 mRNAs in DRG using qPCR method. The results showed that both F4/80 (**Figure 4A**, p <0.05) and CD68 (**Figure 4B**, p <0.01) mRNAs was significantly increased in mice DRG tissue after PTX treatment, but RvD1 had no effect on F4/80 and CD68 mRNAs in PTX-treated mice. We used immunofluorescence technique to label CD68 positive cells in DRG tissue to observe macrophage infiltration. The results were similar to the above qPCR experiment results (**Figure 4C**). These data indicated that the pain-relieving effect of RvD1 on PINP was not through inhibition of macrophage infiltration in the DRG.

### 3.4 RvD1 increases IL-10 production both *in vitro* and vivo

Macrophages exhibit different phenotypes such as M0 (the resting phenotype), M1 (pro-inflammatory phenotype) and M2 (anti-inflammatory/pro-resolution phenotype). These phenotypes are characterized by distinct expression of pro-inflammation cytokines (IL-1β) or anti-inflammation cytokines (IL-10, TGF-β), and are closed related to macrophages functions. PINP involves a strong inflammation condition, and a series of studies have reported that RvD1 could regulate inflammation reaction. We speculate that RvD1 exerts analgesic effect by regulating the function of macrophages.

**Figure 5:**
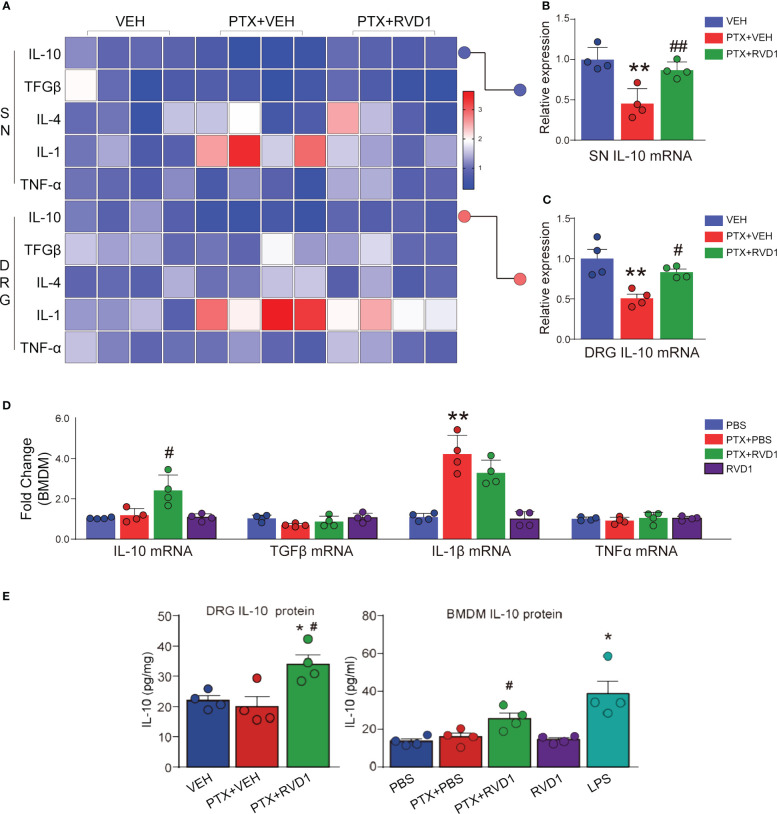
The effect of RvD1 on the expression of inflammatory factors. The inflammatory factors IL-10, IL-4, TGF-β, IL-1β, TNF-α in DRG and SN tissues of mice were detected by qPCR 14 days after PTX injection **(A–C)**. BMDMs were pretreated with 250 nm RvD1 for 3h and then treated with 1 μM PTX for 24 h, the expression of inflammatory factor mRNA was detected by qPCR **(D)**. IL-10 protein was determined by ELISA **(E)**. *p < 0.05, **p < 0.01 vs VEH or CON group; #P< 0.05, ##p< 0.01 vs PTX group. n=4. RvD1, resolvin D1; PTX, paclitaxel; VEH, vehicle.

We detected several inflammation factors mRNAs in mice tissues and BMDM samples. We found that anti-inflammation factor IL-10 was significantly decreased and pro-inflammation factor IL-1β was significantly increased on day 14 in DRG and SN tissues after PTX administration (**Figure 5A**). RvD1 prevention treatment significantly increased IL-10 mRNA both in DRG (**Figure 5B**, p < 0.05) and SN (**Figure 5C**, p <0.01) tissues. However, RvD1 only decreased IL-1β mRNA in SN of PTX-treated mice, but not in DRG (**Figure 5A**).

Furthermore, we cultured BMDM cells to observe the effect of RvD1 on inflammatory factors after PTX treatment. PTX had no significant impact on IL-10 mRNA expression in BMDMs, but RvD1 pretreatment significantly increased IL-10 mRNA in RvD1+PTX group compared with PTX-treated BMDMs (**Figure 5D**, p <0.05). RvD1 pretreatment failed to decrease IL-1β mRNA in BMDMs followed by PTX treatment, which was significantly increased after PTX treatment (**Figure 5D**).

We detected IL-10 protein using ELISA in DRG tissue and BMDMs supernatant. The results showed that IL-10 protein had no significant change in DRGs after PTX treatment, but RvD1 treatment significantly increased IL-10 protein level in PTX-treated mice (**Figure 5E**, p < 0.05). Meanwhile, similar to tissue results, the concentration of IL-10 in the supernatant of BMDMs in RvD1+PTX-treated group significantly increased compared with PTX-treated group (**Figure 5E**, p < 0.05). These data suggested IL-10 was involved in the anti-allodynia effect of RvD1 in PINP.

### 3.5 The analgesic effect of RvD1 on PINP is dependent of IL-10

**Figure 6:**
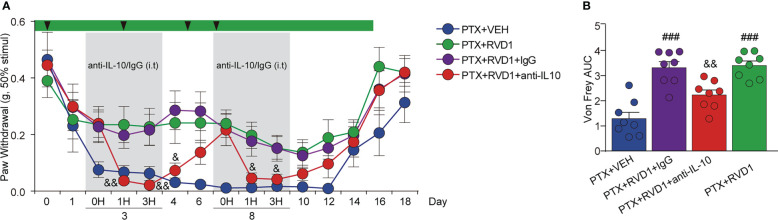
The analgesic effect of RvD1 on PINP is dependent of IL-10. On the 3rd and 8^th^ day of PTX administration, mice were injected (i.t.) with IL-10 neutralizing antibody and IgG (2μg). Mechanical pain threshold was determined using von Frey test **(A)**, AUC analysis was shown in **(B)**. ###p<0.001 vs PTX group; &p<0.05, &&p<0.01 vs PTX+RVD1+IgG group. n=8 in each group. RvD1, resolvin D1; PTX, paclitaxel.

Based on the above results, we speculated that IL-10 was required for RvD1 to attenuate PINP. Next, we used neutralizing antibody specific for mouse IL-10 to examine the contribution of IL-10 in the anti-allodynia effect of RvD1 in PINP mice. IL-10 neutralizing antibody or IgG control antibody was i.t. administrated to target spinal cord cells as well as DRG cells. Mice received a single i.t. injection of the antibodies on day 3 and day 8 after first PTX administration. The i.t. injection of control IgG had no significant impact on mechanical allodynia in RvD1+PTX-treated mice (**Figure 6A**). However, mechanical allodynia threshold rapidly decreased 1 h after i.t. injection of IL-10 neutralizing antibody on days 3 and 8 (**Figure 6A**, p < 0.05). This effect of IL-10 neutralizing antibody could last for 24 hours. The results suggested that IL-10 was required for the analgesic effect of RvD1 on PINP.

### 3.6 RvD1 increases macrophage IL-10 by activating FPR2

**Figure 7:**
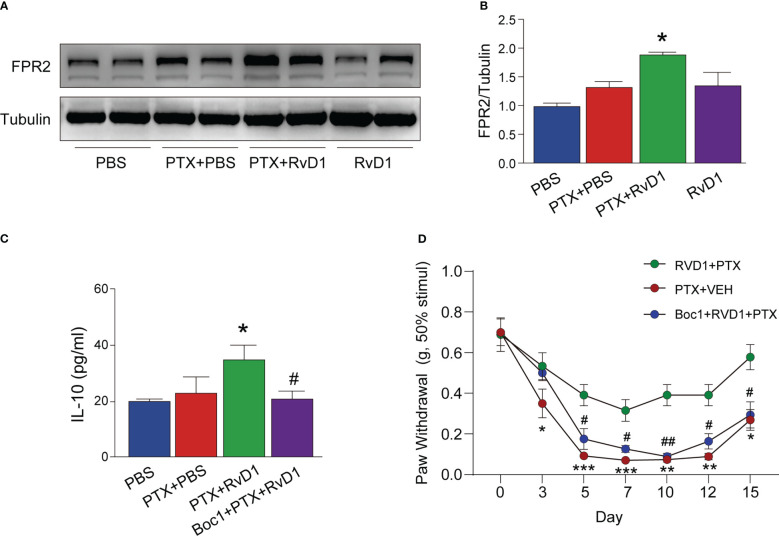
RvD1 promoted the production of IL-10 and its analgesic effect on PINP by activating FPR2 receptor. BMDMs were treated with FPR2 receptor blocker BOC1 (1 μM) for 3 h, RvD1 (250 nm) for another 3 h, and then treated with PTX (1 μM)for 24h. The expression of FPR2 in BMDMs was detected using Western blot **(A, B)**. The IL-10 protein in BMDMs supernatant was detected by ELISA **(C)**. Mechanical pain threshold was determined using von Frey test **(D)**. Boc1 (5 mg/kg/day) was administered intraperitoneally. *p < 0.05, **p < 0.01, ***p < 0.001 vs PTX group; #p<0.05, ##p<0.01 vs RvD1+PTX group. n=4 **(A–C)**, n=8 **(D)**. RvD1, resolvin D1; PTX, paclitaxel.

RvD1 is the endogenous agonist of formyl peptide receptor 2 (FPR2) (21). It has been shown that activation of FPR2 by RvD1 can exert anti-inflammatory effects in many diseases (22, 23). As shown in **Figure 7A**, the FPR2 expression in BMDMs had no significant change after PTX treatment only. However, the FPR2 expression in RvD1+PTX-treated BMDMs was significantly increased compared with PTX-treated group (**Figures 7A, B**). To further confirm the role of FPR2, we used FPR2 antagonist Boc1to observe its effect on IL-10 production. We found that Boc1 abolished the role of RvD1 in promoting IL-10 production (**Figure 7C**, p < 0.05). To test whether FPR2 participated in the relieving effect of RvD1 on PINP, mice were i.p. injected with Boc1. Inhibition of FPR2 signaling eliminated the pain relief effect of RvD1 on PINP (**Figure 7D**). These results suggested the RvD1-FPR2 axis was required for IL-10 production and pain relief effect.

### 3.7 RvD1 attenuates the apoptosis of DRG neurons induced by BMDM conditioned medium

**Figure 8:**
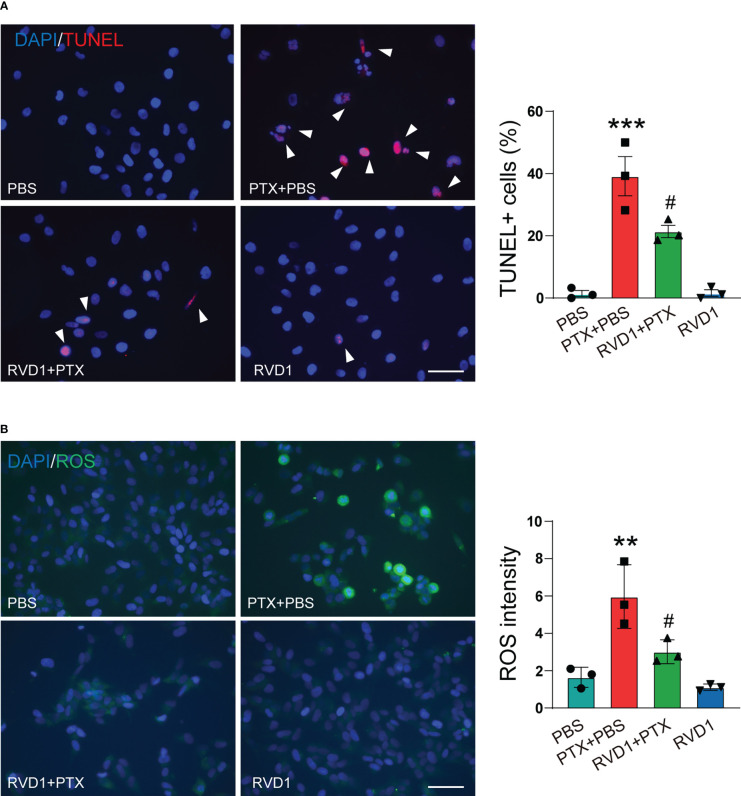
RvD1 attenuates the apoptosis of DRG neurons induced by BMDM conditioned medium. BMDMs were treated with RvD1 (250 nm) for 3 h, and then treated with PTX (1 μM) for 24 h, the culture medium was collected and centrifuged, and the supernatant was taken as the condition medium. The primary DRG neurons were stimulated with the above condition medium for 24 h. The apoptosis of neurons was detected by TUNEL staining **(A)**. The level of ROS was detected by DCFH-DA probe **(B)**. Blue represents nuclei (DAPI staining), red represents apoptotic cells (TUNEL staining), green represents ROS (DCFH-DA staining). **p < 0.01, ***p < 0.001 vs PBS group; ; #p<0.05 vs PTX+PBS group. Scale bar: 100 μm. RvD1, resolvin D1; PTX, paclitaxel.

Since macrophage activation is critical for initiating the neurons apoptosis in several neuropathy diseases (24), we then examined the effect of RvD1-treated BMDMs on DRG neurons apoptosis. The primary mouse DRG neurons were stimulated by different conditioned medium (CM) of BMDM, and the apoptosis neurons were determined using TUNEL staining. First, we prepared 4 groups of BMDMs CM, namely Con CM, PTX CM (treated with PTX only), RvD1+PTX CM (pretreated with RvD1 followed by PTX), RvD1 CM (treated with RvD1 only). As shown in **Figure 8A**, the number of TUNEL-positive DRG neurons significantly increased after the incubation of PTX CM of BMDMs. However, limited apoptosis neurons were observed following incubation of RvD1+PTX CM of BMDMs. The results indicated that RVD1-regulated macrophage could inhibit PTX-induced DRG neurons apoptosis.

### 3.8 The BMDMs RvD1-FPR2-IL-10 axis plays a critical role in DRG neuronal antioxidant damage

Several studies report that oxidative stress contributes for CIPN (25). Under conditions of prolonged oxidative stress, the level of ROS is increased, leading to neuronal cells damage and apoptosis. DRG neurons were stimulated with collected different CMs of BMDMs, using DCFH-DA to detect intracellular ROS. As shown in **Figure 8B**, DCFH-DA fluorescence intensity significantly increased in DRG neurons stimulated by PTX-treated BMDMs CM. However, ROS fluorescence intensity was low when DRG neurons were stimulated by CM from RvD1+PTX-treated BMDMs. The results suggested that RvD1 could alleviate the oxidative stress induced by PTX in neurons by regulating macrophage function.

**Figure 9:**
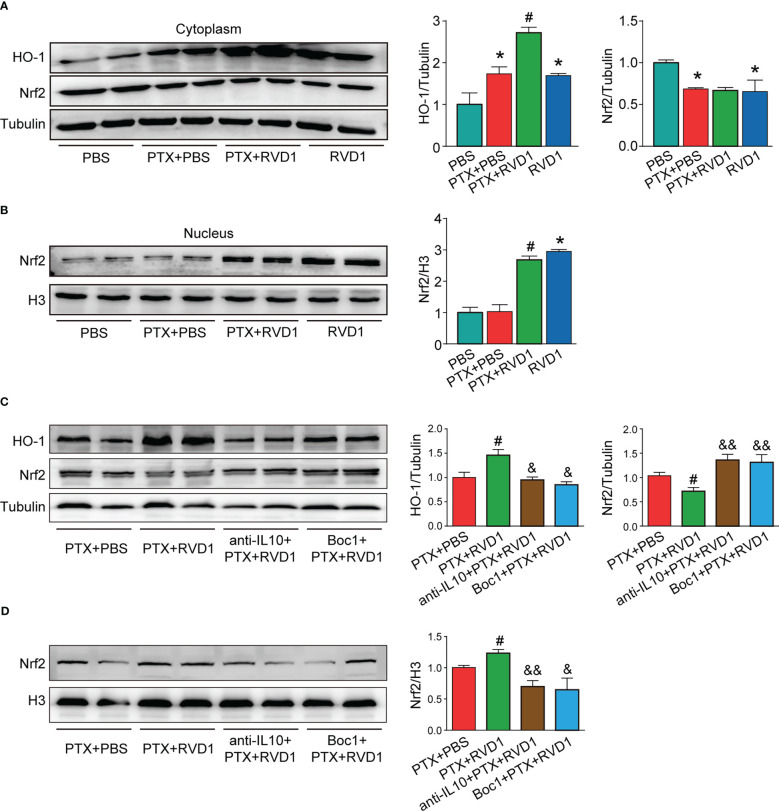
The BMDMs RvD1-FPR2-IL-10 axis plays a critical role in DRG neuronal antioxidant damage. BMDMs were treated with BOC1 (1 μM) for 3 h, then treated with RvD1 (250 nM) for another 3 h, and then treated with PTX (1 μM) for 24 h, then the condition medium was collected. The primary DRG neurons were treated with IL-10 neutralizing antibody, then stimulated with the above condition medium for 24 h. The cytoplasmic and nuclear protein was isolated, and the expression of Nrf2 and HO-1 was detected by Western blot, the band gray was analyzed using Image J **(A–D)**. *P<0.05 vs CON group; #p<0.05 vs PTX group; &p<0.05, &&p<0.01 vs PTX+RvD1 group. n=4. RvD1, resolvin D1; PTX, paclitaxel.

The Nrf2-HO1 signaling pathway is an important pathway for protecting cells from oxidative damage. Although HO1 was increased in DRG neurons after stimulated with PTX CM (**Figure 9A**, p < 0.05), DRG neurons HO1 expression was further increased in RvD1+PTX CM stimulated group (**Figure 9A**, p <0.05). In addition, transcript factor Nrf2 in nucleus had no change in neurons after PTX CM treatment (**Figure 9B**). However, Nrf2 in nucleus was significantly increased in neurons after RvD1+PTX CM treatment compared with PTX CM treatment group (**Figure 9B**, p < 0.05). To further study the role of RvD1-FPR2-IL-10 axis in activation of Nrf2-HO1 signaling pathway, we used FPR2 blocker Boc1 and IL-10 neutralizing antibody to observe the effect on HO1 expression and the sublocalization of Nrf2. We prepared 4 groups of BMDMs CM, namely PTX CM, RvD1+PTX CM, anti-IL-10+RvD1+PTX CM, Boc1+RvD1+PTX CM. As shown in **Figure 9C**, the expression of HO1 in neurons was significantly decreased in anti-IL-10+RvD1+PTX CM and Boc1+RvD1+PTX CM treatment group compared with PTX CM group (p < 0.05). On the other side, blocking FPR2 and IL-10 pathways inhibited Nrf2 nucleus translocation in DRG neurons (**Figure 9D**, p <0.01). The results indicated that RvD1 activated FPR2-IL-10 axis in macrophage contributed for Nrf2-HO1 activation in DRG neurons.

## 4 Discussion

CIPN is a common cause of pain and poor quality of life in cancer patients and cancer survivors. Currently, there is a lack of effective drugs for the treatment of CIPN.

RvD1 belongs to a unique family of lipid mediators called resolvins and has shown significant efficacy in the treatment of inflammation-related diseases. Our previous study showed that another resolvin, RvE1, can attenuate inflammatory pain (18). The main objectives of the present study were to prove whether RvD1 had analgesic efficacy in the mouse PINP model, and to determine whether RvD1 could exert analgesic effect by regulating macrophages. In present study, we found RvD1 attenuated neuropathic pain induced by PTX, and these effects were mainly exerted by regulating the secretion of IL-10 in macrophages. Further, we found that the role of RvD1 in promoting IL-10 secretion by macrophages was dependent on its activation effect on FRP2. In addition, RvD1 could alleviate the damage of PTX to neurons by regulating macrophages *via* Nrf2-HO1 activation. Our study found RvD1 was beneficial for alleviating PINP in mice model, and proposed for the first time that its main mechanism of action was through activation of the RvD1/FPR2/IL-10 axis in macrophages.

RvD1 is an endogenous lipid mediator derived from docosahexaenoic acid (26). Studies have reported that RVD1 has a strong anti-inflammatory effect and is an important mediator in the process of inflammatory resolution (27–29). Our previous study showed that RvD1 attenuated inflammatory pain induced by formalin (18). Other research team found RvD1 displayed potent analgesic properties in irritable bowel syndrome by inhibiting TRPV1 sensitisation (30). Several groups reported that RVD1 could alleviate mechanical allodynia in spinal nerve ligation-induced neuropathic pain (31), noncompressive lumber disk herniation (32), spared nerve injury (33) model. However, the involvement of RvD1 in the resolution of neuropathic pain induced by PTX remains unknown. Interestingly, we found that 12/15-LOX, the key synthetic enzyme of RvD1, decreased in the SN and DRG tissues in PINP mice. Moreover, treatment with exogenous RvD1 significantly promoted pain resolution in PINP. When PINP had been formed, RvD1 still had therapeutic effect. Therefore, these results reveal that RvD1 is benefit to alleviate PINP.

Accumulating evidence suggests that macrophage plays important roles in the pathogenesis and resolution of neuropathic pain (34–36). In most instances, macrophages produce pain by releasing pro-inflammatory mediators such as TNFα and IL-1β, which enhance pain by modulating ion channels (37–39). It reported that PTX could induce macrophages infiltration into the DRG in a time course (8). In present study, we found that mice mechanical pain threshold decreased after intrathecally injected PTX-treated macrophages. In addition, macrophage maker CD68 increased in DRGs in PINP mice. These finding further showed macrophages were involved in PINP. We also found that 12/15-Lox, an endogenous synthase of RvD1, decreased in DRGs in PINP mice. This result indicated RvD1 synthesis was impaired under PINP condition. As a member of resolvins, RvD1 has been reported to act on macrophages to polarize toward a pro-resolution phenotype to alleviate inflammatory response (40–42). In present study, we found macrophages pretreated with RVD1 were able to alleviate PINP. However, RvD1 did not influence the CD68 expression level in PTX-treated mice. This suggested that RvD1 might exert analgesic effects on PINP by regulating macrophage function rather than inhibiting cellular infiltration. However, the exact mechanism was not clear. This requires linking the pharmacological effects of RvD1 with the function of macrophages to explore its role. Usually, macrophages are divided into two categories, M1-like and M2-like. M1-like macrophages, a classically activated phenotype, promote pain by producing pro-inflammatory cytokines and chemokines. In contrast, M2-like macrophages promote tissue repair and pain resolution *via* secreting anti-inflammatory cytokines. Several evidences have demonstrated that chemotherapy induces an increase in peripheral pro-inflammatory cytokines (43), such as TNF-α, IL-1β and IL-6 (44–46). Neuroinflammation has been considered as a potential common driver of CIPN, including PINP. Similar to the previous reports, we also found TNFα and IL-1β increased in SN and DRGs in PINP mice. RvD1 can play a protective role in many diseases by promoting M2 polarization of macrophages. RvD1 can attenuate gouty arthritis pain by reducing leukocyte recruitment and IL-1β production in the knee joint (47). In addition, RvD1 can promote pulmonary inflammation resolution by enhancing M2 polarization. IL-10, IL-4 and TGF-β are classical anti-inflammatory factors secreted by M2-like macrophages. We found RvD1 significantly increased IL-10 and decreased IL-1β in the SN and DRGs tissues in PINP mice. These indicated RvD1 could attenuate PINP by promoting macrophages polarization toward M2 in peripheral neuronal tissues.

It reported that macrophages in DRGs contribute to both the initiation and persistence of neuropathic pain (12), and the endogenous IL-10 from macrophages rather than other cells is required for CIPN resolution (48, 49). It reported that activating the IL-10 signaling pathway by thalidomide or GLP-1 receptor agonist could attenuate neuropathic pain (50, 51). In present study, we found RvD1 increased IL-10 protein level both *in vivo* and in BMDMs. However, blocking IL-10 signal abolished the analgesic effect of RvD1 on PINP. Our results confirmed IL-10 was required for the analgesic effect of RvD1 in neuropathic pain.

RvD1 can bind to FPR2 with high affinity, and is the endogenous agonist for FPR2 (21). FPR2 plays a crucial role in innate immune responses. Previous studies showed FPR2 was expressed on macrophages (52, 53). It reported that FPR2 activation could attenuate inflammatory pain (54, 55). In our study, RvD1 pretreatment increased FPR2 expression in PTX-treated macrophages. Several studies find FPR2 activation regulates macrophage polarization, and promotes inflammation resolution (56, 57). We demonstrated that RvD1 upregulated IL-10 in macrophages *via* FPR2 activation. Our results showed blocking FPR2 by Boc-1 abolished the role of RvD1 in promoting IL-10 production. Meanwhile, Boc-1 abolished the analgesic effect of RvD1 on PINP. These suggested RvD1/FPR2 axis was required for IL-10 production, and was beneficial for PINP.

It is well recognized that apoptosis of neurons in DRG is an important cause of the neuropathic pain in spinal nerve ligation injury and sciatic nerve injury model (58). Also, other studies found neuronal apoptosis in DRG induced by PTX and vincristine contributed to CIPN (59, 60). It is generally considered that activated macrophages play important roles in the initiation of neurons apoptosis in neuropathic pain (24). Consistent with previous research reports, apoptosis was observed in primary DRG neurons after stimulation of condition medium from PTX-treated macrophages. However, apoptosis cell was decreased when the primary DRG neurons were stimulated by condition medium from RvD1+PTX treated macrophage.

In addition, we found ROS in primary DRG neurons decreased in RvD1+PTX-treated macrophage CM group compared with PTX-treated macrophage CM group. Nrf2-HO1 pathway has a critical role against oxidative stress (61). Recently, studies found enhancing Nrf2-HO1 signal can attenuate inflammatory pain by inhibiting MAPK pathway (62), and can alleviate vincristine and paclitaxel induced neuropathic pain (63–65). Nrf2 activation also attenuates chronic constriction injury-induced neuropathic pain *via* induction of PGC-1α-mediated mitochondrial biogenesis in the spinal cord (66). So these evidences indicate that Nrf2 activation is beneficial in chronic pain (67). The Nrf2-based therapy for chronic pain is a promising filed. Previous studies on IL-10 in pain mainly focused on inflammatory response pathways, but in recent years, studies have found that IL-10 can regulate a variety of signaling pathways. IL-10 can upregulate HO-1 expression and play a cardioprotective role in diabetic myocardial infarction (68). IL-10 also promotes HO-1 expression in macrophages, thereby regulating the polarization of macrophagesc (69). We found CM from RvD1+PTX treated macrophage could significantly promote Nrf2 nucleus translocation in DRG neurons. We speculated that IL-10 secreted from macrophages regulated by RvD1 mediates the dialogue between macrophages and neurons. Further, Nrf2 nucleus translocation was inhibited by FPR2 blocker Boc1 and IL-10 neutralizing antibody. Our results showed that RvD1 acted on FPR2 in macrophages to promote the secretion of IL-10, which in turn could activate the Nrf2-HO1 pathway in DRG neurons, exert anti-oxidative and anti-apoptotic effects, and then alleviate neuronal damage.

Our study found RvD1 was beneficial for alleviating PINP in mice model, and proposed for the first time that its main mechanism of action was through activation of the RvD1/FPR2/IL-10 axis in macrophages. In addition, it is worth noting that some studies have reported that RvD1 can control tumor growth (70, 71), suggesting that RvD1 may not affect the therapeutic effect of paclitaxel on tumors when relieving PINP. The results indicated that systemic RvD1 supplementation would be a promising therapy strategy in the treatment of PINP. As for the roles of RvD1 in neuropathic pain induced by other chemotherapy drugs, including oxaliplatin and vincristine, more follow-up studies are needed.

## Data availability statement

The original contributions presented in the study are included in the article/**Supplementary Material**. Further inquiries can be directed to the corresponding authors.

## Ethics statement

The animal study was reviewed and approved by Animal Ethics Committee of Soochow University.

## Author contributions

CS: Investigation - lead, methodology - lead, data curation - lead, formal analysis - equal, writing-original draft - lead, writing-review & editing - equal. J-TZ: Conceptualization - equal, formal analysis - equal, writing-original draft - supporting. FZ: Investigation - supporting, methodology - supporting, equal. DX: Writing-original draft - supporting, writing-review & editing - equal. JP: Formal analysis - equal, writing-review editing - supporting. TL: Conceptualization - equal, data curation - lead, formal analysis - equal, methodology - equal, supervision - equal, writing-original draft - equal, writing-review & editing - equal. All authors contributed to the article and approved the submitted version.
