# Supplementary material for: Resolvin D1/N-formyl peptide receptor 2 ameliorates paclitaxel-induced neuropathic pain through the activation of IL-10/Nrf2/HO-1 pathway in mice
Source: Front Immunol. 2023 Mar 13;14:1091753. doi: 10.3389/fimmu.2023.1091753 (PMC10040838; doi:10.3389/fimmu.2023.1091753)
Supplement: Supplementary file 1 [file Table_1.docx]

Supplementary Table 1.

| Figure | Group, Number | Total Animals |
| --- | --- | --- |
| Fig. 1A-I | VEH (n=8, ICR, male)  PTX (n=8, ICR, male) | N=16 |
| Fig. 1J-K | VEH (n=4, ICR, male)  PTX (n=4, ICR, male) | Same mice as in Fig. 1A-I |
| Fig. 2A-D | VEH (n=8, ICR, male)  PTX (n=8, ICR, male)  RvD1+PTX (n=9, ICR, male) | N=25 |
| Fig. 2E-F | PTX+VEH (n=8, ICR, male)  RvD1+PTX (n=8, ICR, male) | N=16 |
| Fig. 3B-C | PBS (n=6, ICR, male)  MC+PBS (n=6, ICR, male)  MC-PTX (n=6, ICR, male) | N=18 |
| Fig. 3D-E | MC+PBS (n=8, ICR, male)  MC-PTX (n=8, ICR, male)  MC-PTX+RvD1 (n=8, ICR, male) | N=24 |
| Fig. 4A-C | VEH (n=4, ICR, male)  PTX (n=4, ICR, male)  RvD1+PTX (n=4, ICR, male) | Same mice as in Fig. 2A-D |
| Fig. 5A-C | VEH (n=4, ICR, male)  PTX (n=4, ICR, male)  RvD1+PTX (n=4, ICR, male) | Same mice as in Fig. 2A-D |
| Fig. 6 | PTX+VEH (n=8, ICR, male)  PTX+RvD1+IgG (n=8, ICR, male)  PTX+RvD1+anti-IL10 (n=8, ICR, male)  PTX+RvD1 (n=8, ICR, male) | N=32 |
| Fig. 7D | RvD1+PTX (n=8, ICR, male)  PTX+VEH (n=8, ICR, male)  Boc1+RvD1+PTX (n=8, ICR, male) | N=24 |

Supplementary Table 2.

| Antibodies | Company | Cat No. | Website |  |
| --- | --- | --- | --- | --- |
| Rabbit anti-FPR2 | Thermo Fisher | 720293 | https://www.thermofisher.cn/cn/zh/antibody/product/FPR2-Antibody-Polyclonal/720293 | Antigen: Peptide corresponding to Human FPR2 (aa 9-26). This antibody is validated by the company. |
| Rabbit anti-Nrf2 | Proteintech | 16396-1-AP | https://www.ptgcn.com/products/NFE2L2,NRF2-Antibody-16396-1-AP.htm | This antibody was KD/KO validated by the company. |
| Rabbit anti-HO-1 | Proteintech | 10701-1-AP | https://www.ptgcn.com/products/HMOX1-Antibody-10701-1-AP.htm | This antibody was KD/KO validated by the company. |
| Rabbit anti-Tubulin | Proteintech | 11224-1-AP | https://www.ptgcn.com/products/TUBA1B-Antibody-11224-1-AP.htm | On the product website, it is indicated that this antibody has 937 citations of papers that have used it. |
| Rabbit anti-H3 | Beyotime | AF7014 | https://www.beyotime.com/product/AF7104.htm | This antibody is validated by the company. |
